# Dog Bite Epidemiology and Postexposure Practices Among Dog Owners in Some Communities in the Upper West Region of Ghana

**DOI:** 10.1155/jotm/1750043

**Published:** 2026-08-23

**Authors:** Richard Dery Suu-Ire, Amos Sarpong Agyei, Issah Beninmie Alijata, Benjamin Tetteh Mensah, Samuel Asumah, Meyir Yiryele Ziekah, Sylvester Languon, Samuel Otis Bel-Nono, Henry Asigri Abugri, Benjamin Kissi Sasu, Bonodong Zongnukuu Guri, Victor Emmanuel Manieson

**Affiliations:** ^1^ School of Veterinary Medicine, University of Ghana, Accra, Greater Accra Region, Ghana, ug.edu.gh; ^2^ Veterinary Services Directorate, Ghana Ministry of Food and Agriculture, Accra, Greater Accra Region, Ghana; ^3^ Department of Animal Laboratory Sciences, University of Ghana, Accra, Greater Accra Region, Ghana, ug.edu.gh; ^4^ Wildlife Division, Forestry Commission of Ghana, Accra, Greater Accra Region, Ghana; ^5^ University of Ghana West African Centre for Cell Biology of Infectious Pathogens, Accra, Greater Accra Region, Ghana; ^6^ Ghana Armed Forces, Accra, Greater Accra Region, Ghana; ^7^ Ghana Atomic Energy Commission, Accra, Greater Accra Region, Ghana, gaecgh.org; ^8^ African Field Epidemiology Network, Kampala, Central Region, Uganda

**Keywords:** community survey, dog bites, Ghana, postexposure practices, rabies, upper west region

## Abstract

**Background:**

Rabies remains a neglected tropical disease in northern Ghana, where dog bites are common, and postexposure prophylaxis access is limited. Community‐level data on bite epidemiology and health‐seeking practices are scarce.

**Methods:**

A cross‐sectional survey was conducted among 370 dog‐owning households (1824 individuals) in Lawra and Jirapa municipalities, Upper West Region, using a multistage sampling approach. Three submunicipalities were randomly selected from each municipality, after which households were selected using systematic sampling. We captured lifetime dog bite history, postexposure practices and rabies prevention behaviours.

**Results:**

Overall rabies awareness was high (Jirapa 97.6% and Lawra 77.0%). Lifetime household‐level dog bite prevalence was significantly higher in Jirapa (44.1%) than in Lawra (20.5%). Postbite care was suboptimal: 38.4% of victims in Jirapa first sought traditional remedies compared to 17.2% in Lawra. Death after a bite was reported in 33.3% of Jirapa victims and 16.4% of Lawra victims. Family members reported symptoms suggestive of rabies in some of these cases; however, none were clinically or laboratory confirmed. Higher education was positively associated with safer practices (*β* = 0.78, *p* = 0.008), while initial reliance on traditional healers was inversely associated (*β* = −1.12, *p* = 0.005).

**Conclusions:**

Dog bites and probable rabies deaths are underreported in these municipalities. The heavy reliance on traditional medicine and low formal health‐seeking highlight critical barriers to rabies elimination. Strengthening community‐based surveillance, integrated traditional healer referral, and targeted radio‐based health education in local languages are urgently needed.

**Summary**
• What is already known
◦ Dog‐mediated rabies continues to pose a serious public health threat across sub‐Saharan Africa, where the vast majority of human cases result from bites inflicted by infected domestic dogs. In rural and resource‐limited settings, access to timely postexposure prophylaxis (PEP) remains severely constrained, and disease surveillance systems are often weak or fragmented. Under these conditions, delays in seeking formal healthcare and reliance on traditional remedies following dog bites are widespread practices that contribute substantially to preventable human deaths.
• What this study adds
◦ This study contributes community‐level epidemiological data on the burden of dog bites and postexposure practices in two rural municipalities of northern Ghana. It reveals marked spatial heterogeneity, with dog bites more than twice as prevalent in Jirapa compared to Lawra, and demonstrates that reliance on traditional remedies and delays in seeking formal healthcare remain common, particularly in Jirapa. The findings confirm that children bear a significant share of the bite burden and identify higher education as an important determinant of safer postexposure practices. Furthermore, the study highlights that lethal and nonreporting responses to suspected rabid dogs persist, undermining both surveillance and control efforts in these communities.

## 1. Introduction

Dog‐mediated rabies remains one of the most lethal yet preventable zoonotic diseases worldwide. More than 95% of human rabies deaths result from dog bites, with an estimated 20,000 deaths occurring annually in Africa, over half of which affect children under 15 years of age [1, 2]. Despite the availability of effective vaccines and PEP, rabies continues to cause substantial mortality in many low‐ and middle‐income countries due to inadequate surveillance systems, limited access to healthcare and persistent sociocultural practices that influence treatment‐seeking behaviour [3, 4].

In Ghana, rabies remains endemic and continues to pose a significant public health challenge. Dogs are responsible for over 90% of reported animal rabies cases in the country between 2014 and 2022 [5]. Although Ghana has adopted the global target of eliminating dog‐mediated human rabies by 2030, progress towards this goal remains uneven, particularly in rural and underserved regions where veterinary and healthcare infrastructure is limited. In such settings, dog bites are often managed outside formal healthcare systems, and the use of traditional remedies following suspected rabies exposure remains common [6, 7]. These practices significantly increase the risk of fatal outcomes because rabies is almost invariably fatal once clinical symptoms appear.

Understanding the epidemiology of dog bites and community responses following exposure is, therefore, essential for designing effective rabies prevention strategies. Beyond vaccination coverage, rabies control requires insight into who is bitten, where exposures occur, how communities respond after bites and the factors that influence healthcare‐seeking behaviour. Such information helps identify behavioural and systemic barriers to timely PEP administration and effective rabies surveillance.

However, empirical data on dog bite burden, postexposure practices and community responses to suspected rabid animals remain limited in northern Ghana. Lawra and Jirapa municipalities in the upper west region represent rural settings characterised by close human–dog interaction, subsistence livelihoods and constrained access to veterinary and health services. These conditions may increase the risk of rabies exposure and limit effective postexposure management.

This study, therefore, investigated the epidemiology of dog bites and postexposure practices among dog‐owning households in Lawra and Jirapa municipalities. Specifically, the study estimated the prevalence of dog bites, described the demographic characteristics and outcomes of bite victims, examined actions taken following dog bites, assessed preventive behaviours and responses to suspected rabid dogs and identified sociodemographic determinants of safer postexposure practices. By examining both exposure patterns and behavioural responses, this study provides community‐level epidemiological evidence to inform rabies prevention and control strategies within a One Health framework in rural Ghana.

## 2. Methods

### 2.1. Study Area and Setting

The study was conducted in Lawra and Jirapa municipalities in the upper west region of Ghana. Both municipalities are predominantly rural, with high dependence on agriculture, limited access to veterinary and public health infrastructure and close human–dog interactions, making them appropriate settings for a rabies study. The combined population of the two municipalities is approximately 150,000 [8]. The geographical location of the study area is shown in Figure 1.

### 2.2. Study Design

A community‐based cross‐sectional survey was conducted among dog owners in Lawra and Jirapa municipalities between 23rd and 29th September 2020. The study population consisted of households that owned dogs within the two municipalities. A multistage sampling approach was employed. In the first stage, submunicipalities were selected using simple random sampling. In the second stage, households within the selected submunicipalities were selected using systematic sampling.

### 2.3. Sample Size Calculation

A sample of 370 dog owners aged 18 years or older, each from a different household, was used. The sampling frame comprised the total number of households (8,300) in the six selected submunicipalities. Sample size was determined using Cochran’s [9] formula for finite populations:

(1) n0=Z2p1−pe2.

In this equation, *n*_0_ represents the initial sample size, *Z* is the standard normal deviate corresponding to the desired confidence level (1.96 for a 95% confidence level), *p* is the estimated proportion of the population (0.5 for maximum variability) and *e* denotes the desired margin of error (0.05). Substituting these values into the formula, the initial sample size is *n*_0_ = 384.

(2) n0=1.962×0.510.5−0.052=3.84160.25×0.0025=384.16384≈.

Because the study population was finite (*N* = 8300 households), the initial sample size was adjusted using Cochran’s finite population correction formula:

(3) n=n01+n0−1/N,

where *n* denotes the adjusted sample size, *n*_0_ is the initial sample size obtained from equation above and N is the total number of households in the sampling frame (8,300). Substituting these values into the finite population correction formula gave

(4) n=3841+3841−/8300=3841+3838300/=3841.04614=367.07367≈.

The calculated minimum sample size was 367 participants. To compensate for potential nonresponse and incomplete questionnaires, the target sample size was increased to 370 participants.

### 2.4. Selection of Submunicipalities

A simple random sampling technique was used to select three submunicipalities from each of the two main municipalities. All submunicipalities were assigned numbers, and three numbers were drawn randomly by simple balloting. The three selected numbers represented the submunicipalities included in the study.

### 2.5. Household Selection

After the submunicipalities were selected, a systematic sampling technique was used to select households. The sampling interval of 22 was calculated by dividing the total number of households (8,300) by the target sample size (370). To ensure a random start, the first household was selected by spinning a bottle at a central landmark, identified with the assistance of local opinion leaders. Thereafter, every 22nd household was selected for questionnaire administration.

### 2.6. Handling of Nonresponse

When a selected household was temporarily unoccupied, the adjacent household was selected. Similarly, if a household had no dog owner in the preceding 5 years, it was replaced by the nearest adjacent household. Field teams made up to three repeat visits (daytime, evening and weekend) before excluding a household. Households where no eligible respondent could be reached after three visits were excluded.

### 2.7. Respondent Selection Within Households

In each selected household, the owner of the dog(s) was invited to participate. If the primary dog owner was unavailable, the next eligible adult household member involved in dog care was selected. If there was more than one eligible dog owner in a household, a simple random sampling technique (balloting) was used to select one respondent.

### 2.8. Data Collection

A structured questionnaire was used to collect data on sociodemographics, dog ownership, lifetime dog bite history, postbite actions, knowledge of rabies and rabies prevention practices. For bite‐related deaths, we recorded whether the victim died after the bite and asked about key rabies symptoms using the local terms “gbandiru” (mad dog disease) and “saabie” (inability to swallow water). Higher education was defined as education beyond senior high school, including vocational, technical, diploma and university‐level education.

## 3. Composite Scores

### 3.1. Two Composite Scores Were Constructed for Analysis

Dog‐Bite Postexposure Practice (PEP) Score (0–4): This score summed the following safe actions after a bite: (i) wound washing with soap and water, (ii) seeking care at a health facility, (iii) completing the recommended vaccine course and (iv) avoiding traditional remedies. Each item was scored one if performed and 0 if not, giving a range of 0–4.

Rabies Prevention and Control Practices Score (0–4): This score summed the following prevention practices: (i) confining owned dogs, (ii) vaccinating owned dogs against rabies annually, (iii) reporting suspect rabid dogs to veterinary or health authorities and (iv) participating in community clean‐up to reduce stray dogs. Each item was scored one if performed and 0 if not.

Internal consistency was assessed using Cronbach’s alpha (PEP Score = 0.68; Prevention Score = 0.71).

### 3.2. Data Analysis

Data were analysed using Stata 17.0. Descriptive statistics were used to summarise variables. Differences between municipalities were tested using chi‐square tests for categorical variables and *t*‐tests for continuous variables. Predictors of the Dog‐Bite PEP Score and Rabies Prevention and Control Practices Score were examined using multivariable linear regression, adjusting for municipality of residence. Municipality of residence was included as an adjustment variable in all regression models to account for differences between the two municipalities.

### 3.3. Handling of Clustering

Households were nested within communities and municipalities. Because only two municipalities were included in the study, municipality of residence was included in all regression models as an adjustment variable to account for differences between municipalities rather than as a clustering variable. Village‐level clustering could not be modelled because community identifiers were unavailable for all households, and this limitation is acknowledged when interpreting the findings.

### 3.4. Ethical Approval

Ethical approval was granted by the Catholic University of Ghana Institutional Review Board, and administrative clearances were obtained from the Lawra and Jirapa Municipal Health Directorates. Written or witnessed verbal informed consent was obtained from all participants before data collection.

## 4. Results

### 4.1. Characteristics of Respondents and Dog Ownership

The 370 households comprised 1824 individuals. Most respondents were male (62.7%), married (78.1%) and farmers (64.3%). Only 12.7% had attained higher education. The average number of dogs per household was 2.4, and only 16.8% of dogs were reportedly vaccinated against rabies in the preceding year.

### 4.2. Knowledge and Awareness

Awareness of rabies as a disease was higher in Jirapa (97.6%) than in Lawra (77.0%, *p* < 0.001). However, knowledge of transmission through a bite from an infected dog was limited (Jirapa 52.4% and Lawra 38.3%). Many respondents attributed rabies to spiritual causes or ‘poisoning’.

### 4.3. Dog Bite Burden

Lifetime household dog bite prevalence was 44.1% in Jirapa and 20.5% in Lawra. Bite victims were predominantly children < 15 years (58%).

### 4.4. Postexposure Practices and Outcomes

In Lawra, 63.3% of bite victims visited a hospital first, while in Jirapa, only 29.5% did so. Conversely, 38.4% of Jirapa victims first sought help from a traditional healer (“bangre‐ma”) or used herbal preparations. The proportion of victims reported to have died after the bite was 33.3% in Jirapa and 16.4% in Lawra. Family members recalled that several decedents experienced symptoms suggestive of rabies, including aimless running, hydrophobia and agitation; however, these reports were based on recall and were neither clinically nor laboratory confirmed. Regarding actions upon seeing a suspected rabid dog, 23.8% of Jirapa respondents initially took ‘other’ actions: 9.2% informed the earth priest (Tindana), 5.1% temporarily relocated children, 4.3% attempted to kill the dog themselves and 3.2% attributed the event to a curse and did nothing.

### 4.5. Predictors of Safer Practices

In the multivariable model, higher education was significantly associated with a higher Dog‐Bite PEP Score (*β* = 0.78, 95% CI 0.21–1.35, *p* = 0.008), while traditional healer’s first consultation was inversely associated (*β* = −1.12, CI −1.89 to −0.35, *p* = 0.005). Sex, age, occupation and municipality were not independent predictors of the PEP Score. For the Rabies Prevention Score, only dog vaccination status was a significant positive predictor.

## 5. Discussion

This study provides important community‐level epidemiological evidence on dog bite burden and postexposure practices in two rural municipalities of northern Ghana. Nearly one‐third of dog‐owning households reported a history of dog bite, with a significantly higher prevalence in Jirapa than in Lawra. This marked spatial heterogeneity suggests localised differences in dog population density, roaming patterns or human–dog interactions. Similar intraregional variations have been documented in other African settings, where dog ecology and community‐level management practices strongly shape exposure patterns [3, 6].

Postexposure practices revealed critical vulnerabilities in rabies prevention. Although health facility attendance was relatively common, particularly in Lawra, reliance on traditional remedies was substantially higher in Jirapa. This pattern is consistent with the findings from Ghana and other sub‐Saharan African countries, where sociocultural norms, perceived cost barriers and limited access to formal healthcare drive traditional medicine use after potential rabies exposure [7, 10, 11]. Given that rabies is almost invariably fatal once clinical symptoms appear, such practices significantly elevate the risk of preventable deaths [1].

Children constituted a substantial proportion of bite victims, accounting for 58% of reported cases. This finding aligns with global evidence indicating that children bear a disproportionate burden of dog‐mediated rabies [2]. Increased vulnerability among children is often attributed to behavioural factors, closer physical interaction with dogs and reliance on caregivers for healthcare‐seeking decisions.

A particularly concerning finding was the high proportion of reported deaths following dog bites, especially in Jirapa. Although these deaths were not clinically or laboratory confirmed and therefore cannot be definitively attributed to rabies, family members reported that several individuals experienced symptoms suggestive of rabies before death, including hydrophobia and agitation. These findings should be interpreted cautiously, as other conditions such as tetanus or severe wound infections may also have contributed to mortality. Nevertheless, the reported fatalities highlight the need for strengthened surveillance, improved access to PEP and systematic follow‐up of bite victims in these communities.

Responses to suspected rabid dogs further illustrate structural weaknesses in rabies control. Although over half of respondents indicated that they would report to an animal health officer, lethal actions and passive responses remained common. Such practices undermine laboratory confirmation, compromise surveillance accuracy and may perpetuate unrecognised transmission cycles. Similar surveillance challenges have been described across Africa, where fear‐driven killing or nonreporting of suspect animals weakens rabies control efforts [3, 4].

The finding that higher education was a significant predictor of safer postexposure practices highlights the role of health literacy in shaping behavioural responses. Individuals with higher education levels may better understand rabies severity and the importance of prompt PEP initiation. However, the absence of demographic predictors for rabies control behaviour suggests that unsafe or nonreporting responses are widespread across population groups, emphasising the need for broad community‐based interventions.

Collectively, these findings demonstrate that rabies risk in rural northern Ghana is shaped not only by exposure frequency but also by behavioural responses following exposure and by surveillance gaps. Achieving Ghana’s goal of eliminating dog‐mediated human rabies by 2030 will require a strengthened One Health approach that integrates sustained mass dog vaccination, improved access to affordable PEP, community education emphasising immediate wound washing, engagement of traditional healers as referral partners and robust surveillance systems capable of tracking bite outcomes [1, 2]. Without addressing these behavioural and reporting gaps, preventable rabies deaths may remain undetected within vulnerable rural communities.

## 6. Limitations

Several limitations should be considered when interpreting these findings. First, the study relied on participants’ recall of lifetime dog bite events, which is subject to recall bias and may have resulted in under‐ or overreporting of bite occurrences. Because the survey collected lifetime bite history only, annual incidence rates could not be estimated, and comparisons with studies that used shorter recall periods should be interpreted with caution. Future studies should incorporate a defined recall period, such as 1 year, to generate more accurate estimates of recent dog bite burden and facilitate comparisons across settings.

Secondly, the Dog‐Bite Postexposure Practice (PEP) Score demonstrated modest internal consistency (Cronbach’s alpha = 0.68). Although this level is considered acceptable for exploratory community‐based research, it falls below the conventional threshold of 0.70 and suggests that the scale may benefit from further refinement and validation in larger populations.

Thirdly, postbite mortality data were based on self‐ or family‐reported outcomes without laboratory confirmation or formal verbal autopsy procedures. Consequently, reported deaths following dog bites may have resulted from rabies, tetanus, sepsis or other causes, limiting the ability to attribute mortality specifically to rabies.

Fourthly, clustering of households within communities could not be fully accounted for because community‐level identifiers were unavailable for all households. Although regression models were adjusted for municipality of residence, residual clustering may have influenced the precision of some estimates.

Finally, the cross‐sectional design limits causal inference. Associations observed between respondent characteristics and postexposure practices should, therefore, be interpreted as correlational rather than causal relationships.

## 7. Conclusion

This study demonstrates that dog bites represent a substantial yet underrecognised public health challenge in Lawra and Jirapa municipalities, with significant spatial variation in bite prevalence and postexposure practices. While rabies awareness was widespread, this knowledge did not consistently translate into safe postbite actions. Reliance on traditional healers and incomplete reporting of suspect rabid dogs remain critical gaps that undermine rabies prevention efforts in these communities.

The finding that higher educational attainment was associated with safer postexposure practices highlights the potential of targeted health education, yet the absence of demographic predictors for rabies control behaviour suggests that unsafe responses cut across all population groups. This reinforces the need for universal, community‐wide interventions rather than approaches focused solely on specific subgroups.

The reported occurrence of deaths following dog bites, particularly in Jirapa, highlights important surveillance and case‐management challenges. Although these deaths were neither clinically nor laboratory confirmed and should be interpreted cautiously, they underscore the need for strengthened community‐based surveillance, systematic follow‐up of bite victims and improved access to PEP. Strengthening community‐based case tracking and integrating bite outcome monitoring into existing health information systems are essential steps towards more accurate burden estimation.

Moving forward, a coordinated One Health response is required. Local health and veterinary authorities should collaborate with community leaders, traditional healers and local radio networks to deliver culturally appropriate rabies education in Dagaare, promote immediate wound washing with soap and water and improve access to affordable PEP. Mass dog vaccination campaigns, coupled with enforcement of dog confinement bye‐laws, remain the cornerstone of sustainable rabies control. Without sustained investment in these preventive and surveillance measures, Ghana’s target of eliminating dog‐mediated human rabies by 2030 will remain out of reach for communities in the upper west region.

## Author Contributions

Richard Dery Suu‐Ire, Amos Sarpong Agyei, Bonodong Zongnukuu Guri and Meyir Yiryele Ziekah conceived the study and developed the research design.

Issah Beninmie Alijata, Benjamin Tetteh Mensah, Victor Emmanuel Manieson, Sylvester Languon, Samuel Otis Bel‐Nono, Henry Asigri Abugri, Samuel Asumah and Benjamin Kissi Sasu participated in field data collection and manuscript review. All authors contributed to data interpretation.

## Funding

This study received financial support for Field Data Collection and Community Engagement Activities was Provided by the Rabies in West Africa (Riwa‐Ghana) Project.

## Disclosure

All authors approved the final manuscript. The funder had no role in the study design, data analysis, decision to publish or preparation of the manuscript.

## Conflicts of Interest

The authors declare no conflicts of interest.

## Supporting Information

Additional supporting information can be found online in the Supporting Information section.

## Supporting information


**Supporting Information** Figure 1: Map of Ghana showing the upper west region and the study areas (Lawra and Jirapa municipalities).

## Data Availability

The datasets generated and analysed in the current study are available from the corresponding author upon reasonable request.
