# Supplementary material for: Dog Bite Epidemiology and Postexposure Practices Among Dog Owners in Some Communities in the Upper West Region of Ghana
Source: J Trop Med. 2026 Aug 23;2026:1750043. doi: 10.1155/jotm/1750043 (PMC13501008; doi:10.1155/jotm/1750043)
Supplement: Supplementary file 1 [file JOTM-2026-1750043-s001.docx]

**Figure 1. Map of Ghana showing the Upper West Region and the study areas (Lawra and Jirapa municipalities).**
